# Assessment of Stress in the Soil Surrounding the Axially Loaded Model Pile by Thin, Flexible Sensors

**DOI:** 10.3390/s21217214

**Published:** 2021-10-29

**Authors:** Krzysztof Żarkiewicz, Waleed Qatrameez

**Affiliations:** Faculty of Civil and Environmental Engineering, West Pomeranian University of Technology, Piastów 50a, 70-311 Szczecin, Poland; wqatrameez@gmail.com

**Keywords:** tactile pressure sensor, color film pressure sensor, pile load test, stress in soil, laboratory investigation

## Abstract

Foundation piles transfer the applied vertical load to the surrounding soil by skin friction and base resistance. These two components induce stress in the soil. The load transfer is still not fully recognized, and some pile load tests analyses have raised many doubts. The present paper aimed to measure the stress levels during pile load tests in laboratory conditions. This research examined the possibilities of using thin, flexible sensors in measuring the stress in soil. Two sensors were used: tactile pressure sensor with mapping system and color film pressure sensors with digital analyzing. Calibration and preliminary tests of the sensors have been described. This calibration proved that this kind of sensor could measure the stress in the soil in laboratory conditions. The results of stress distribution in the soil, shown as pressure maps, have been presented. Significant stress changes were observed in pile load tests. Rough and smooth piles were compared in the analyses. Stress distribution was the result of simultaneous interaction of pile skin and base. The knowledge about stresses surrounding the pile allows us to carry out a deeper analysis of the pile–soil interaction.

## 1. Introduction

The tactile sensor has recently become crucial in various technologies. One of these technologies is engineering, specifically for detecting objects and their weight under outside effects such as loads and forces. This analysis helps engineers and designers to understand how the parts fit each other and follow their performance during the testing period [1]. The tactile pressure sensors and pressure indicating film both have advantages and constraints. This is particularly relevant when endeavoring to give accurate and significant information which relies upon the kind of physical constraints of the estimated system and required data.

Tactile sensor technology empowers to measure stresses at an enormous number of points in nearness, in this manner considering a practical normal stress distribution. The innovation was initially evolved at MIT’s Artificial Intelligent Laboratory by Hillis [2] and Purbrick [3], invigorated by dental application. A firm named TEKSCAN has improved the sensor and its usage for clinical and engineering applications. TEKSCAN holds proprietary and patented sensor technology [4]. Tactile pressure sensors are used for pressure measurements, specifically dynamic pressure mapping. In addition to the software related to tactile pressure, the sensor also includes different devices for analysis, making it a significant piece of the overall pressure system. On the other hand, the negative side of utilizing tactile sensors is related to the extended setup times that are needed to guarantee that calibration has been carried out appropriately. For accurate measurements, the calibration of the sensor ought to be completed, utilizing materials that are the same as those utilized in the application. Paikowsky and Hajduk [5] described the main application of the technology for geotechnical engineering.

Pressure measurement film is used to measure the interface pressure between two surfaces, and is the fundamental technique that shows shading, creating material with tiny microcapsules layered over a polyester material. Each color microcapsule breaks when pressure is applied. In other words, unique microcapsules crack at various pressures, and the more pressure is applied to a given area, the more capsules get broken. The slenderness of the film permits it to be utilized in a variety of applications to catch pictures of pressure profiles. Another advantage of pressure film sensor film is that no electronics are joined, which permits the film to gather pressure distribution data without worrying about wires and costly hardware. Pressure film works well to have a peak pressure between objects. It is more complicated to utilize them in different processes, such as estimating variations below peak pressure and endeavoring to comprehend the adjustment of pressure after some time. Moreover, when repeated tests are required, more pieces need to be cut and set, which takes additional time and increases the cost of materials.

Granular materials are involved in different discrete units. These materials exist in our daily life, including food (for example, sugar, salt), mechanical powders (for example, coal, glass), and chemicals materials, in addition to the soils under each construction and tunnels. Previously, the estimations of stresses acting inside or at the limit of a granular mass were assessed primarily using generally large load cells and gave a limited number of estimations. In addition, the utilization of load cells is complicated because of the interference of the estimating device with the load distributions inside the material and the measurements of results. The slender uniform structure of the tactile pressure sensors significantly diminishes the obstruction brought about by the estimating components and considers a more exact recording of the stresses in granular materials. Paikowsky and associates were the first to utilize tactile pressure sensors for geotechnical applications. Paikowsky and Hajduk [5] reported on a complete series of sensor tests in granular media. They presumed that the tactile pressure sensor system gives typical normal stress measurements in granular soil to a decent level of exactness. Moreover, they showed that sensor estimations are delicate to load rate, creep, and hysteresis after dumping and gave test information to evaluate these impacts. They utilized these sensors for visual observation and measurement of aerial stress distribution under a rigid strip footing [6] and vertical stress distribution beneath a pile of sand [4]. The tactile sensor has additionally been utilized by different analyses for different applications, for example, measuring the vertical stress transmitted from railroad tracks [7], sensing changes in the vertical stress of pipelines that are displaced along the side [8], and the calibration of tactile pressure sensors for measuring stress in soils [9]. Previous studies focused on stress measurements at the soil interface. The calibration of the tactile sensor has been known to be essential for estimation precision [5]. Some other studies aimed at soil shear strength determination in the laboratory and in situ tests and highlighted particle contact as very important in soil particle breakage and strength behavior [10,11]. This paper presents laboratory measurements of stress in the soil surrounding the pile by using advanced technology represented in tactile sensors (Tekscan) and pressure film sensors (4 LW and 5 LW) to evaluate and calibrate the soil stress during a pile load test in laboratory conditions.

Piles transfer the vertical applied load by the interaction with the surrounding soil. Piles gain the load capacity by the skin friction and base resistance. The mechanisms of resistance mobilization of the pile’s skin and base are completely different, but both cause the stress to increase in the soil. The stress in the soil balances the force that comes from the pile until the failure criterion is reached. It is worth adding that the local failure on the pile’s skin does not mean that the pile achieved the ultimate load capacity because the base may still have the reserve of capacity. Different pile settlements are needed to induce the failure on the skin and base of the pile. Pile load capacity is usually interpreted by the ultimate base resistance and skin friction. Some of the theories allow calculating the pile load capacity based on soil properties. However, it still does not fully recognize the relationship between resistances mobilization and changing soil stress. This was the starting point of the research and the main aim of the presented paper.

**Figure 1:**
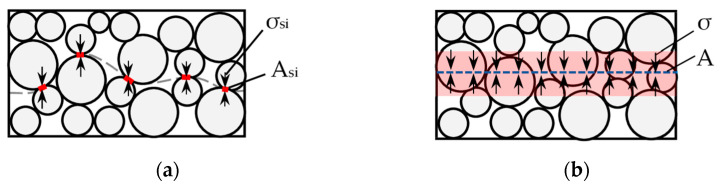
Stress distribution in soil. (**a**) Real distribution; (**b**) equivalent distribution.

Stress in the soil is transferred by grains with very small dimensions (e.g., for sand, only 0.05–2.00 mm). In this case, the contact stresses between the grains are huge and may achieve 150 MPa when the plastic strain phase is achieved [12]. The average stress in soil and the contact stress between soil grains is presented in Figure 1.

The distribution in the soil can be calculated with Equation (1):

(1) $$\sigma A=\sum \sigma_{si}A_{si}\;,$$

where σ—average stress in the cross-section, kPa; *A*—area of the cross-section, m^2^; $\sigma_{si}$—contact stress between soil grains, kPa; $A_{si}$—area of soil grains contact, m^2^.

The diameter of the contact area of two soil grains is described by Equation (2) [12]:

(2) $$d={\left[\frac{12\left(1-v^{2}\right)}{E}\cdot \frac{R_{1}R_{2}}{R_{1}+R_{2}}F\right]}^{1/3},\;\mathrm{mm}$$

where v—Poisson ratio; E—Young modulus, GPa, $R_{1},R_{2}$—radius of soil grain No. 1 and No. 2, respectively, mm; F—force transferred between grains, N.

Equation (2) allows calculating the contact stress in the soil due to the average stress in a soil sample.

**Figure 2:**
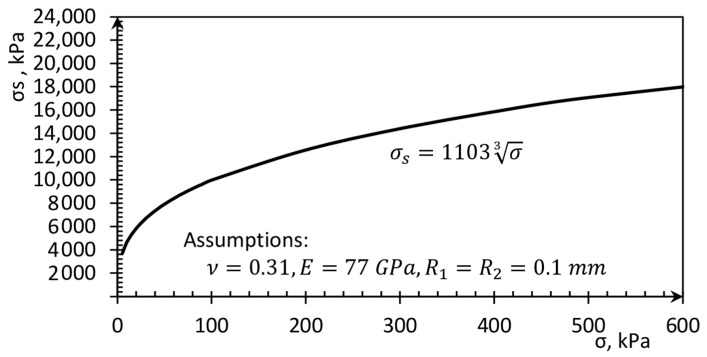
Contact stresses in soil grains versus average stress in a soil sample.

The contact stress is huge in comparison with the average stress, as is presented in Figure 2. In geotechnical engineering, the contact stresses are usually neglected, but they may significantly impact the soil interaction with other materials. In combination with soil with smooth material, soil grains may cause microcavities which increase the friction conditions. In the case of steel smooth pile, the stress on the steel–soil interface equals 1 MPa, which may induce cavities with a depth of 0.09 mm, resulting in a rough surface formation and improvement of friction conditions.

Furthermore, based on Figure 2, it can be noticed that very small average stresses can induce contact stresses which are several hundred times greater than the average value. The contact stress is very difficult to measure because of the very small interaction area. Measurement and DEM simulations of the contact soil pressure was also described in [13,14].

The other phenomenon of stress distribution in the soil is the stress concentration, which is developed in the form of chains of increased stress. The chains are especially visible in coarse-grained soils such as sand and gravel, and they form a network of chains of contact forces [15,16].

Generally, in geotechnical engineering, soil stresses are not considered stresses between particles but rather at a bigger scale than the average stress in the analyzed area, as in research presented in [7,9,17,18].

## 2. Materials and Methods

Stress in soil depends on the forces distributed by the particles. Any sensor which is placed in the soil to measure the pressure changes the conditions of force distribution; thus, the sensor should be small, thin, and flexible, and should deform together with the soil without any obstacles. A wide range of sensors is used in geotechnical engineering; among them are tactile and color film pressure sensors [19].

In this paper, two kinds of sensors are presented which were used to measure contact stresses. First is the tactile pressure sensor, which has 1936 measuring points and allows measuring interface pressure changes. Second is the pressure color film, which can precisely measure pressure distribution and record the pressure peak during the test. Both were used in laboratory tests to investigate the stress changes in the soil surrounding the loaded pile.

### 2.1. Tactile Pressure Sensor

**Figure 3:**
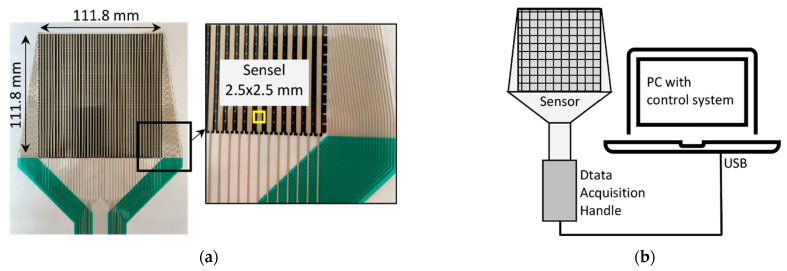
(**a**) Photo of the tactile sensor with a zoom on one sensel. (**b**) Scheme of the measuring system.

The tactile pressure sensor used in the laboratory test consists of two polymeric sheets with pressure-sensitive semiconductive ink printed on each sheet. Typically, the ink is imprinted on lines on one sheet and in columns on the other. At the point when the two sheets are squeezed together, a framework of sensing areas is shaped. The active area for measuring was equal to 0.0125 m^2^ (dimensions: 111.8 × 111.8 mm) and thickness was only 0.102 mm. The cross of rows and columns on the sensing area is called a sensel. From one sensel, information about the normal force and the localization was gained. The sensor has 1936 sensels, distributed over an area equal to 6.25 mm^2^. The large density of sensels allows measuring the pressure distribution with good accuracy. The photo and scheme of the sensor are presented in Figure 3. Two ranges of tactile sensors were used: 0–41 kPa and 0–345 kPa [20].

### 2.2. Pressure Color Film Sensor

The pressure color film can be characterized as a pressure demonstrating sensor film that might be utilized to uncover the distribution and magnitude of pressure between any two contacting or impacting surfaces. This sensor can easily measure pressure equilibrium and distribution on any size. The sensor consists of two sheets: the first is a polyester base with a micro-encapsulated color-forming layer, and the second is a polyester base with a color-developing layer. Connecting the two layers activates the sensor. After the sensor is placed between two compressed surfaces, the microcapsules are broken and react with a color-developing agent [21]. During the test, the maximum pressure is recorded by the intensity of the color. The film sensor can be cut into various shapes from rolls with 320 mm.

**Figure 4:**
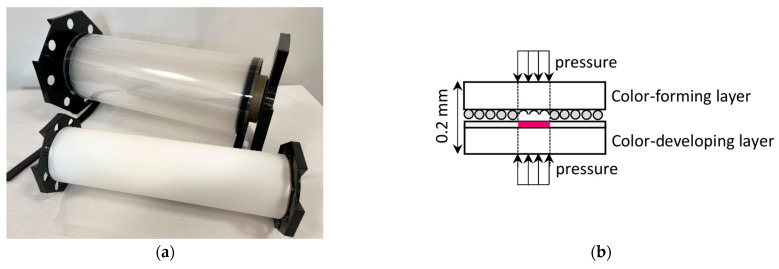
(**a**) Photo of the color film sensor. (**b**) Measuring method.

In the laboratory test, two kinds of pressure were used, Ultra Extreme Low Pressure (5 LW), with a pressure range of 6–50 kPa, and Extreme Low Pressure (4 LW), with a pressure range of 50–200 kPa. The thickness of the two-sheet sensor is only 0.2 mm. The photo and scheme of the measuring method are presented in Figure 4.

The advantage of using the sensor is the ability to cut any shape, its small size, its flexibility, and no accompanying equipment such as cables and data loggers. The above features allow measuring pressures in hard-to-reach places such as the soil surrounding the pile, aimed in this paper and other applications [22].

### 2.3. Instrumented Model Piles

Piles transfer the applied load into the soil by the skin friction and base resistance. Foundations piles in engineering practice have the diameter and length usually in the range of 0.2 m–1.5 m and 5 m–30 m, respectively. Due to the layered soil and strong diversity of soil parameters, it is hard to analyze the stress in the soil on a real scale. Therefore, the small scale of piles is used in the presented research. Laboratory conditions allow to maintain the high repeatability of results and simplify the analysis. Therefore, model tests are still valuable [23]. In laboratory research, three model foundation piles were used. The piles have different geometry and surfaces, as is presented in Table 1.

**Figure 5:**
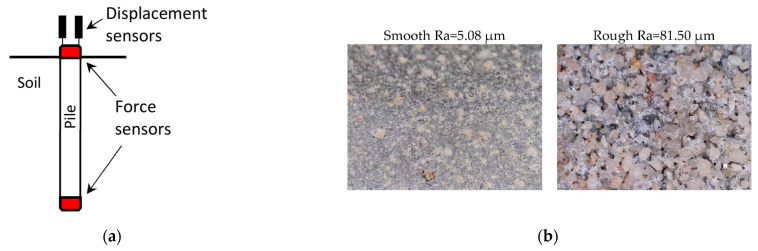
(**a**) Scheme of the instrumented pile. (**b**) Photo of different surfaces of piles.

Two different surfaces allow distinguishing two different schemes of failure. The rough surface makes sufficient connections with soil grains, so the failure occurs far from the pile skin. A smooth pile surface weakens the pile–soil interface connection. Earlier failure due to insufficient roughness can lead to failure before the increase in stress in the soil due to dilatation may occur described in point 2.4. This paper used the roughness description qualitatively rather than quantitatively to distinguish only the different phenomena described above and presented in Figure 5b.

Piles were instrumented in strain gauge force sensors in the head and at the base of the pile as is presented in Figure 5a. The range of a measured force is 0–10 kN, and accuracy is 0.01 kN. Optoelectronic encoder sensors were used with a range of 0–50 mm and an accuracy of 0.005 mm to measure the pile displacement.

### 2.4. Soil

The soil used in a laboratory test is medium sand with grain uniformity index equals 3.04, while the minimum and maximum soil porosity index are equal to 0.452 and 0.776, respectively. Medium sand has less than 4.35% silt and clay fraction and no gravel, cobbles, and boulders fraction. The maximum grains are 2 mm, and the substitute aggregate of grains together with the smaller one account for 50% is equal to 0.3 mm.

**Figure 6:**
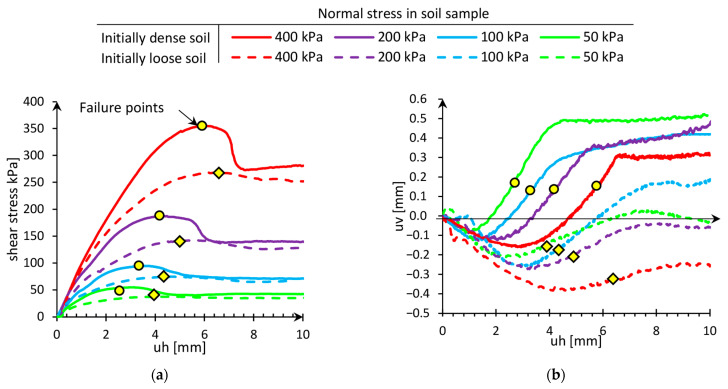
The behavior of soil in a direct shear test. (**a**) Shear stress versus horizontal displacement. (**b**) Changing the height of the sample versus changing horizontal displacement.

The strength of soil was tested in a shear box due to the ISO [24] standard requirements. The soil was tested in four normal stresses, i.e., 50, 100, 200, and 400 kPa, and in two different initial densities, i.e., loose and dense, due to the requirements [25] presented in Figure 6.

Figure 6a presents the relationship between the shearing stress and horizontal displacement of the upper part of the shear box. Dense and loose soil is described by the continuous and dashed lines, respectively. In dense soil shearing tests, the peaks of stresses are observed, which are more prominent than in a loose state. After the peak, the stress in dense soil decreased to achieve the critical shear stress close to the stress achieved in shear tests of loose soil. This phenomenon is known as the hardening and softening behavior of soil, and it was also observed in field conditions [26]. The failure points refer to the maximum shearing resistances, which are achieved in different displacements. The higher the normal stress in soil, the greater horizontal displacement needed to cause the failure—the results in Figure 6b present dilation and contraction during shearing. The initial contraction was observed in loose and dense soil, but greater values were in a loose state.

Additionally, the failure points refer to the centers of the straight range of dilation. These relationships may have a significant influence on stress forming in the main part of the research: pile load test with flexible sensors. In the direct shear test, the normal stresses were maintained constant, but in the pile load test, the surrounding soil is partially blocked by the other soil particles. The dilations observed before the maximum shearing resistance may cause an increase in the normal stress in natural conditions and increase the strength of the soil.

## 3. Sensors Calibration

### 3.1. Calibration of Tactile Sensors

**Figure 7:**
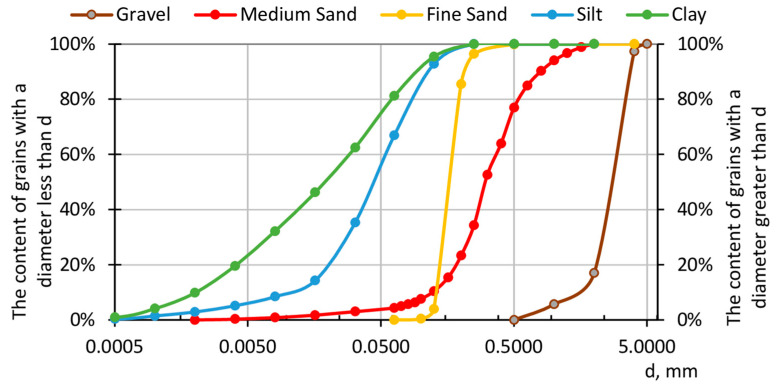
Cumulative grain distribution curves of the tested soils.

Tactile sensors were calibrated in a testing machine. The main aim of the calibration was to check the difference in pressures obtained for different soils with different soil particles size. This phenomenon was also investigated by Paikowsky et al. [4]. As was mentioned in [27], calibration of the tactile sensor should be thoroughly considered for measuring the surfaces with different stiffness because of different outcomes, but in this research, only the stresses between the same material were considered. In the calibration, five different soils were used, i.e., gravel, medium sand, fine sand, silt, and clay, with the medium value of the particle size distribution *d*_50_ equal to 2.770, 0.303, 0.166, 0.044, and 0.019, respectively. Soil grain distribution curves were measured using the sieve method for gravel or sands and laser diffraction to measure particle size distribution for silt and clay. Figure 7 presents the cumulative grain distribution curves of the tested soils.

**Figure 8:**
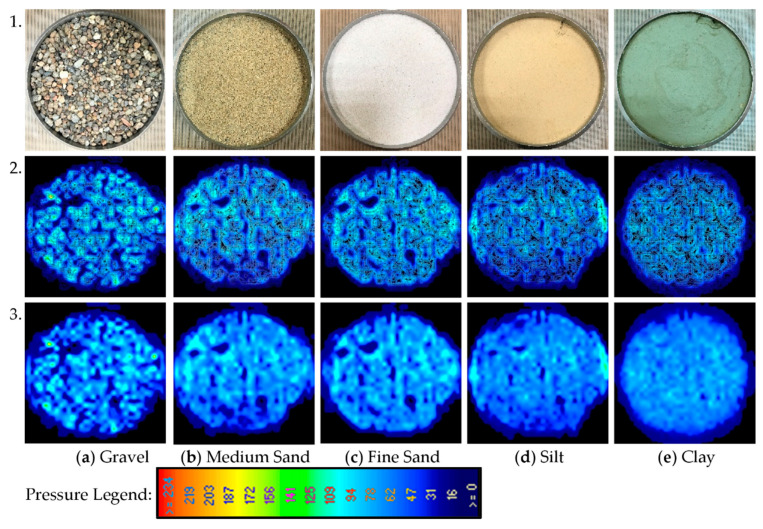
Stress maps were obtained by the tactile sensor for different soils. Row 1. Photo of soil, Row 2. Contour stress, Row 3. Average stress. (**a**) Gravel; (**b**) Medium Sand; (**c**) Fine Sand; (**d**) Silt; (**e**) Clay.

In calibration, the assumed pressure was applied to the sample, and the stress read by the tactile sensor was compared with the applied value of 50 kPa. Then outcomes were implied in the calibration settings of the software. Figure 8 presents the photo of used soils and results of stress distribution. It can be seen that the average contact stress was equal to the applied value, but the measurements from the sensels spread from 0 kPa to almost twice the applied pressure. The contact stress was more homogeneous for fine particles (clay, silt) than coarse particles (sand, gravel). The pressure maps presented in Figure 8, Row 2 indicate that the stress concentration is much greater than the size of the particles. For example, the spacing between the maximum values on the pressure maps for medium sand is approximately equal to 7 mm. In contrast, the equivalent diameter of the medium sand is equal to 0.3 mm.

**Figure 9:**
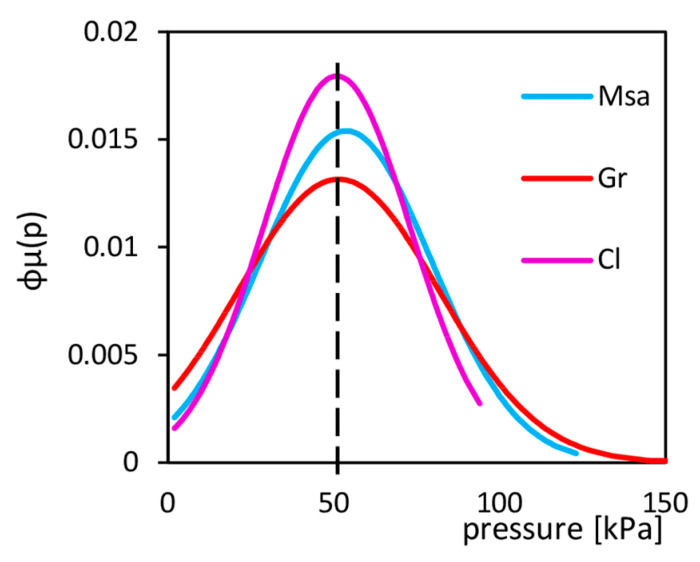
The stress probability distribution functions for soils with different grain sizes, Msa—Medium sand, Gr—Gravel, Cl—Clay.

The diversity of stress makes a difference in the interface quality because, at the same force, the more significant stresses can cause the micro recesses in materials and then improve the friction coefficient. The comparison of stress concentration presents in Figure 9. The average applied pressure was equal to 50 kPa. For gravel, there were many points with pressure equal to 0 and exceeding 100 kPa, which was not observed for clay, where most of the points indicated 50 kPa.

### 3.2. Calibration of Color Film Sensors

After the calibration of the tactile sensor, both tactile and color film sensors were used for simultaneous calibration. In this calibration, both 4 LW (50–200 kPa) and 5 LW (6–50 kPa) sensors were calibrated with tactile sensors with a range of reading 0–41 kPa and 0–345 kPa. In this calibration, only one soil was used from the previous calibration, i.e., medium sand. This sand was also used in the main tests.

The calibration procedure was as follows:

1. Install the base consisting of a steel plate with dimensions of 100 × 100 mm and thickness of 5 mm and put the extruded polystyrene layer with a thickness of 15 mm.
2. Make a thin layer of sand and install the tactile and color film sensor.
3. Install the soft dilation ring and a steel ring, and fill it with soil.
4. Install the steel piston, which transfers the applied load into the soil.
5. Apply the assumed pressure and wait 5 min to align the readings.
6. Unload the sample, save the reading from the tactile sensor, and scan the color film for digital analysis.

The dilation ring described in point 3 was used to avoid the stress concentration described widely in [28].

**Figure 10:**
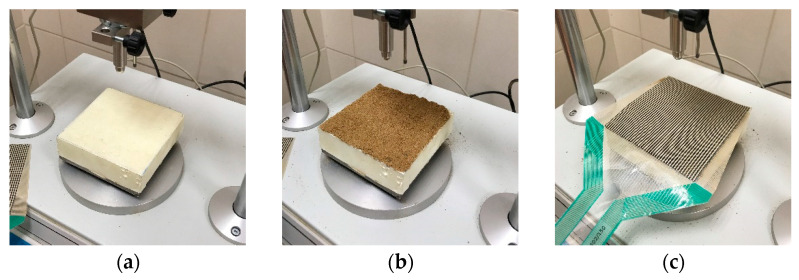

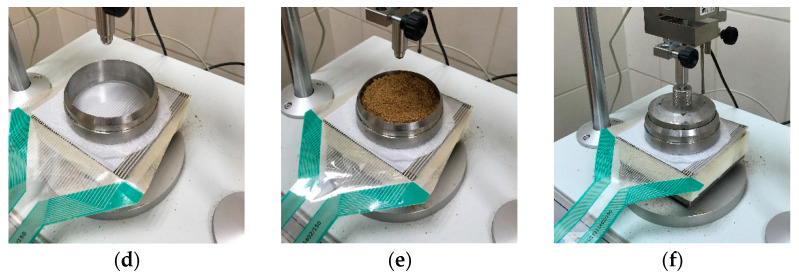
Calibration of tactile and color film sensors. (**a**) Steel plate (thickness 5 mm) and extruded polystyrene (thickness 15 mm) layer. (**b**) Layer of sand. (**c**) Tactile sensor. (**d**) Color film sensor and steel ring with flexible and soft dilatation ring placed between the steel ring and sensor. (**e**) Filing the ring with sand. (**f**) Placing the steel piston and starting the test.

**Figure 11:**
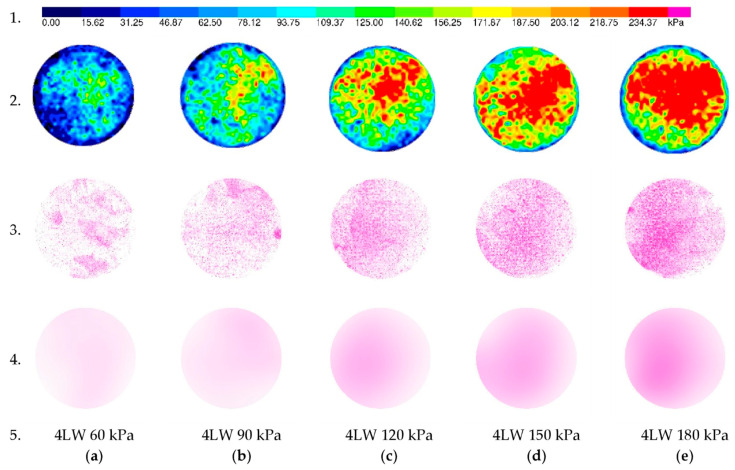
Comparison of contact stresses obtained from tactile and color film sensor 4 LW due to different applied stresses. Row 1. Pressure Legend, Row 2. Pressure from tactile sensor results, Row 3. Raw outcomes from color film sensors; Row 4. Average stress on the surface obtained from color film sensors; Row 5. Type of color film sensor and applied stress; (**a**) 60 kPa; (**b**) 90 kPa; (**c**) 120 kPa; (**d**) 150 kPa; (**e**) 180 kPa.

**Figure 12:**
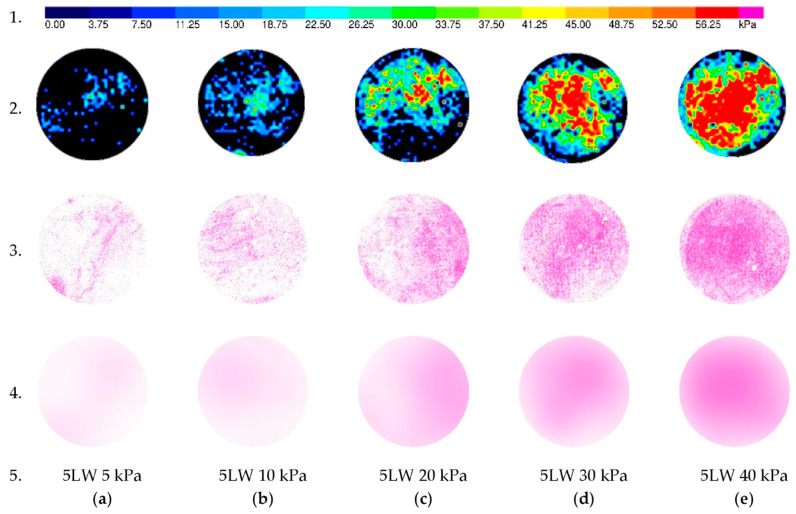
Comparison of contact stresses obtained from tactile and color film sensor 5 LW due to different applied stress. Row 1. Pressure Legend, Row 2. Pressure from tactile sensor results, Row 3. Raw outcomes from color film sensors; Row 4. Average stress on the surface obtained from color film sensors; Row 5. Type of color film sensor and applied stress; (**a**) 5 kPa; (**b**) 10 kPa; (**c**) 20 kPa; (**d**) 30 kPa; (**e**) 40 kPa.

Figure 10 shows the calibration procedure described above. The calibration tests were carried out individually for different assumed pressures. The calibration results are present in Figure 11 and Figure 12.

During the calibration, the most problematic was removing the sample to obtain an undisturbed measurement for color pressure films, especially 5 LW. Just a soft touch of 5 LW color film causes the measurement. That was why it so essential to avoid any touching which did not come from the sample.

Figure 11 demonstrates the results of each of the tactile sensors and color pressure films. The first row shows the legend of the tactile sensor. The second row presents five results from tactile sensors. As Figure 11 shows, the load increased from left to right 60–180 kPa, respectively. Tactile sensors provide pressure maps that include total force, pressure distribution, peak pressure, the center of force, and forces in different areas. The third row demonstrates the density of the purple color from color pressure film 4 LW. As Figure 11 shows, the density of the color increases forwardly with applied load (the first one was under a load of 60 kPa). The effect of color is light comparing with the second circle, which refers to 90 kPa. The second is lighter than the third circle and so on until the last circle is darker under an applied load of 180 kPa. The fourth row is the average color shading of the third row obtained from the image analysis software. Pressure maps obtained from the calibration are not homogeneous, as is presented in Figure 7. It was the intended action gained by the not-uniform density of the soil. The aim was to obtain not only the average value but also the pressure distribution on the contact surface.

The results in Figure 12 are similar to Figure 11, but with different colors distribution and film 5 LW under the applied load range (5–40 kPa). The second row shows the results from the tactile sensors. As mentioned above, in the 5 LW, the color starts from blue to red simultaneously with increased load. The results from the color films appear in the third row. As in the 4 LW presented in Figure 11, the fourth row is the average of the third row.

Overall, the shape of the color effects on the pressure films is close to the shape of the load effect on soil from the tactile sensors test.

The border effect noticed in the results presented in Figure 11 and Figure 12 might be caused by the dilative ring with very low stiffness compared with steel ring or soil and caused the stress reduction. Additionally, the total measured force in the sensor due to the stress integral is equal to the applied load because of the stress concentration in the central part of the sample. This effect did not affect the calibration process. The dilation ring aims to avoid forces transferred by the steel cover ring. Therefore, the authors stated that this effect should not affect the main test and soil stress measurements in practice.

The presented calibration allows creating the pressure scale for the 4 LW and 5 LW color films, with the soil used in the primary research. Discrepancies in stress distribution are acceptable. Therefore, both 4 LW and 5 LW were qualified to use as additional sensors in the pile load test. A comparison of the accuracy of tactile sensors and color film sensors in other applications was presented by Bachus et al. [29].

### 3.3. Compaction Influence on Initial Stress in Soil

The color pressure film sensors, in contrast to tactile sensors, measure only the peak stress. Both sensors were placed during the laboratory stand preparation. The soil compaction process could leave a trace of initial stress that was impossible to remove before the main test. This is the reason to investigate the influence of soil compaction on the initial stress in the soil and the initial readings on the sensors.

**Figure 13:**
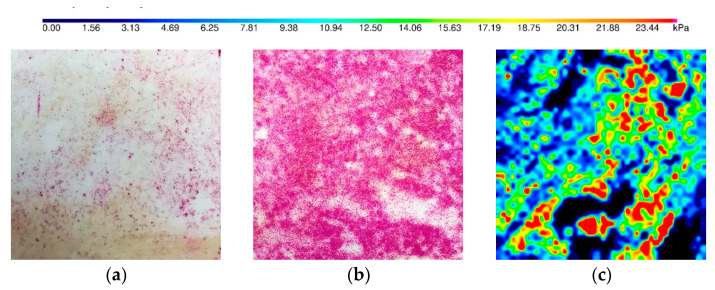
Pressure in the soil below the 10 cm thickness compacted layer measured by: (**a**) 4 LW color film sensor; (**b**) 5 LW color film sensor; (**c**) tactile sensor.

The soil was placed into the chamber layer by layer and compacted using a steel plate dropped from the assumed height. One layer with 10 cm thickness was compacted by dropping the plate ten times. The weight, initial fall high, and plate area equaled 14.4 kg, 0.05 m, and 0.118 m^2^, respectively. In this way, the applied energy during the compaction process maintains the same level. The two sheets of color film sensors (5 LW and 4 LW) and the tactile sensor were placed below the 5 cm and 10 cm layers of soil to check the preliminary reading on sensors. After that, the plate was dropped into the soil layer 14 times. Then, both the color film and tactile sensor were excavated. The tactile sensor allows measuring the pressure versus time, while the color film sensor can record only the maximum pressure which occurred during compaction. The results of the compaction influence are presented in Figure 13.

Figure 13 indicates that the compaction process induces many points with pressure excess of 20 kPa. Some points were measured by the 4 LW sensor, which could measure stresses greater than 50 kPa. This is the reason to reject using 5 LW and then use 4 LW, which was not significantly affected by the compaction process and then might be ready to measure the stress caused by the pile. The tactile sensor measured the stresses due to the time and was not affected by the compaction process. The pressure map presented in Figure 13c is only one frame, which refers to the peak of one chosen dynamic impact of the steel plate.

**Figure 14:**
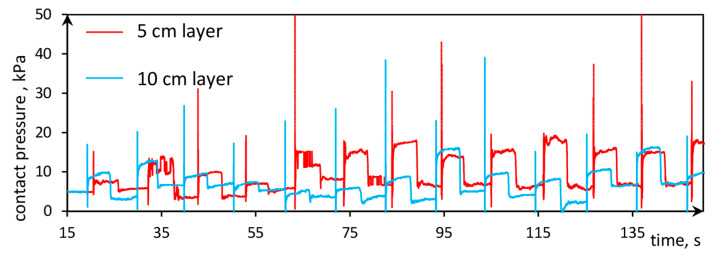
Contact pressure below the 5 and 10 cm compacted layer obtained by the tactile sensor.

**Figure 15:**
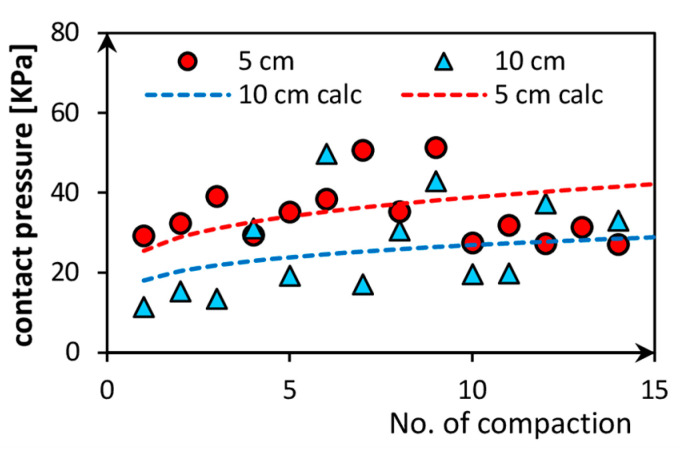
Contact pressure versus the number of compaction falls.

The tactile sensor allows to simultaneously measuring the pressure during the compaction process. The system can record the outcomes with a frequency of 100 Hz. This frequency in some steps was insufficient to record the very short peak of pressure, which takes less than 0.01 s. Figure 14 presents that after the peak, which is analyzed deeper in Figure 15, the stress caused by the dynamic compaction is not immediately reduced because the plate was lying on the compacted layer surface. After 5 s, the plate was taken and the stress reduced to the initial value. It is worth adding that the reduction is not equal to the stress, resulting from the static influence of the lying steel plate because the static pressure is only equal to 1.25 kPa (resulting from the weight of the steel plate), while the reduction was greater than 5 kPa.

The rapid amplitudes presented in Figure 14 refer to the dynamic fall of the steel plate. After the plate induced the dynamic force, the stress in the soil decreased to zero due to the pressure wave induced in the soil. The peaks were not recorded in every dynamic compaction because the measurement frequency equals 0.01 Hz. Capturing all measuring points to gain better precision and repeatable peak values was not possible.

Figure 15 demonstrates the influence of compaction falls on the maximum contact pressure during compaction. Mayne and Jones described the impact of stresses during dynamic compaction of soil [30]. Contact pressure increases due to the number of compaction fall, but the pressure reaches the stabilize level off the pressure, and then the change is almost imperceptible. Dashed lines in Figure 15 are the functions obtained from the energy law of the compaction process described by Equation (3):

(3) $$\sigma =\sqrt{\left(ghmE_{0}\right)/\left(tA\right)}\;,\;\mathrm{Pa}$$

where: g—gravity acceleration, which equals 9.81 m/s^2^; h—plate drop height, which equals 0.05 m; m—the weight of steel plate, which equals 14.4 kg; $E_{0}$—elasticity modulus of soil in the range 540–1200 Pa, t—thickness of soil layer, which equals 0.05 or 0.10 m; A—area of the plate, which equals 0.118 m^2^.

Two effects might explain the irregular behavior of the measured contact pressures. First, the initial contact area in every compaction step was not the same due to the declination of the plate to the soil surface. Second, the stress that was induced during compaction of the 5 cm soil layer was higher than the observed in compaction of the 10 cm layer, especially at the beginning of compaction, because of the stiffness of soil, which increased faster in the case of a thin layer.

## 4. Laboratory Pile Load Tests

**Figure 16:**
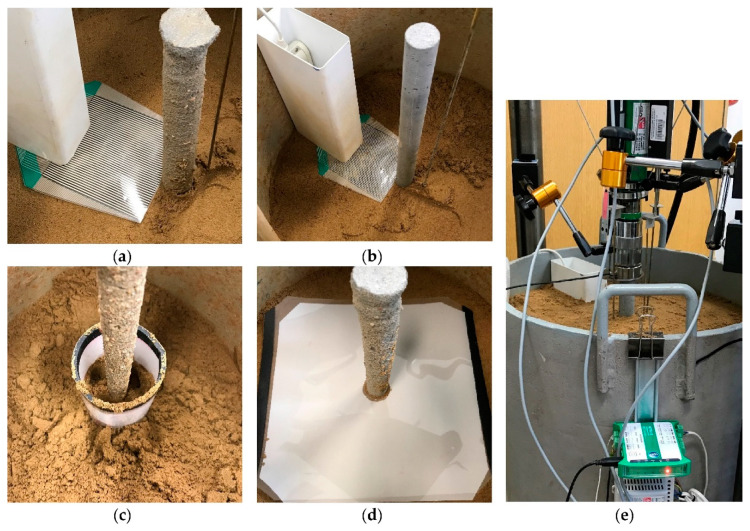
Photos of the laboratory stand with different measurement schemes. (**a**) The tactile sensor placed horizontally at the half of the pile depth P1.R.0.5H.TS. (**b**) The tactile sensor placed horizontally at the pile depth P2.S.H.TS. (**c**) The color film pressure sensor placed vertically around the pile P6.R.CFS. (**d**) The color film pressure sensor placed horizontally P5.R.CFS. (**e**) Laboratory stand prepared for the test.

**Figure 17:**
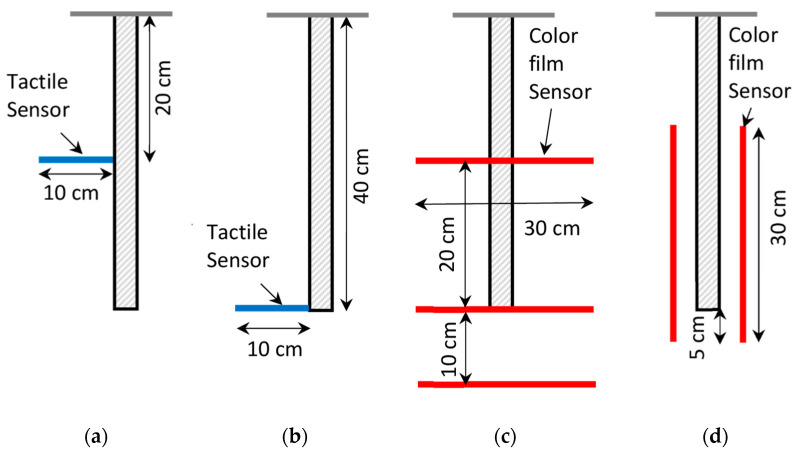
Schemes of the tests. (**a**) The tactile sensor placed horizontally at the half of the pile depth P1.R.0.5H.TS and P1.S.0.5H.TS. (**b**) The tactile sensor placed horizontally at the pile depth P3.S.H.TS and P4.R.H.TS. (**c**) The color film pressure sensors placed horizontally P5.R.CFS. (**d**) The color film pressure sensor placed vertically at different depths P6.R.CFS.

In the laboratory investigation, the instrumented piles were used to transfer the vertical load to the soil. Both sensor and pile were placed during soil compaction. Sensors were placed in different localizations, as is described in Table 2 and Figure 16 and Figure 17.

Piles were loaded in assumed load steps by a hydraulic cylinder. In each step of the test, when the constant force was maintained, the settlement, base resistance, and pressure outcomes in the case of the tactile sensor using were measured. The measured values were recorded at a frequency of 1 Hz. The soil response to the force that comes from the pile was not immediate because of the elastoplastic behavior of soil, which was especially visible in the force close to the ultimate pile load capacity. Thus, the next load step took place after the settlement stabilizes off, and the requirement of stabilization 0.02 mm/min was met. After every test, the soil was excavated, and sensors and piles were carefully taken out. The color film sensors were then scanned to be analyzed using computer graphics software.

The duration of one test was roughly equal to 50–90 min, which in terms of the amount of data gives 6–10 million outcomes for tactile sensors and 3–5 thousand outcomes for force and settlement sensors. The number of outcomes from color film depends on the scan resolution, equal to 600 dpi in this research.

Sensors were placed in the soil at different localizations, as is presented in Figure 16 and Figure 17.

Sensors were placed in the soil at the localizations presented in Figure 16 and Figure 17. Figure 16a,b show the tactile sensor with a rough and smooth pile placed at half of the pile depth and at the pile depth level, respectively. Sensor touched the pile skin at one edge. The data acquisition handle needed to be installed close to the sensor. Therefore, an additional cover should be installed to protect the handle from the soil. Figure 16c,d present the color film censor placed in vertical and horizontal directions, respectively. The soil was carefully placed on the sensor and between sensor and pile, and was then compacted after achieving the required thickness of the layer.

Obtaining the natural state of relative density without influencing initial reading created many difficulties, especially for low pressures. The 5 LW color film sensor is very sensitive, and any touch may cause unintentional measurements. It was the reason to check the compaction influence on the initial reading. After obtaining the results of these preliminary tests of compaction influence, we used only the 4 LW color film sensor, which has a range of measurement beyond the compaction influence, as is presented in Figure 13. However, the 5 LW color film sensors helped prove the compaction influence. The higher ranges of pressures in soil should increase the precision of measurements because it allows neglecting the initial stress caused by the compaction process and causes a better soil–sensor connection.

## 5. Results

The applied load *N*_2_, skin friction *T*, base resistance *N*_1_, pile settlement *s*, and the pressure maps from the tactile sensor were recorded during each test at a frequency of 1 Hz t. Pile response as the settlement to the applied load was initially linear, but every next stage causes an increase in the increments of settlement versus the applied load. This relationship is commonly known as the pile settlement curve. The ultimate pile load capacity refers to the applied load that goes to the asymptote. This asymptote sets out the pile load capacity, which is impossible to exceed because the pile settlement should achieve infinity from a mathematical point of view. Both slips of soil on the pile skin and failure in the soil below the pile base occur in the above situation.

**Figure 18:**
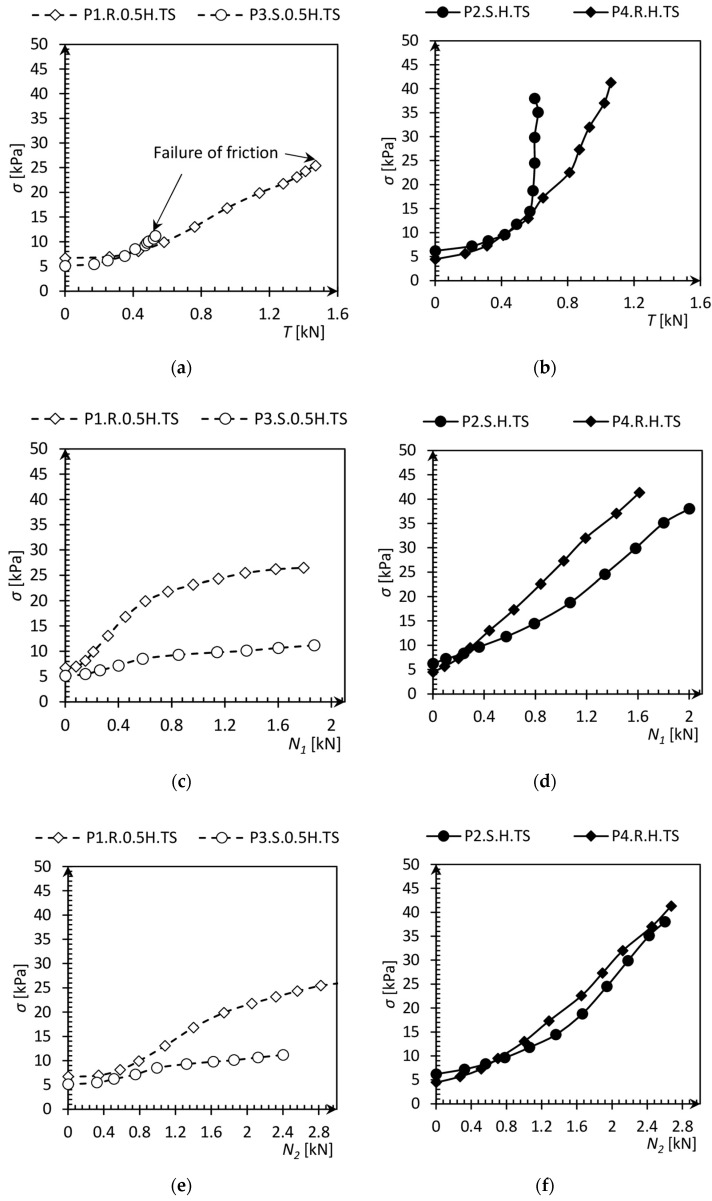
Pressure changes due to the skin friction *T*, base resistance *N*_1_, or applied load *N*_2_. Dashed lines mean pressure at the half of pile length level, solid lines mean pressure at pile base level. (**a**,**b**) Vertical stress versus skin friction. (**c**,**d**) Vertical stress versus skin friction. (**e**,**f**) Vertical stress versus applied load.

Figure 18 shows the relationship between the vertical stress measured by the tactile sensors placed in two different localizations and the resistances or applied load. Stress in the soil presented in Figure 18a increases approximately linearly from the initial geostatic stress to the maximum stress refers to the failure of pile skin friction. This does not mean that the pile had reached the ultimate capacity, but it does mean that the further increases did not change soil stresses. It indicates that stress changing at the level of half of the pile depth was caused only by the friction between the pile skin and surrounding soil. Moreover, it can also be noticed that increasing stress did not go hand in hand with skin friction mobilizing at the beginning of the diagram in Figure 18. This could be caused by the contraction phenomena described in point 2.4. After that, the stress increasing before shearing was caused by the dilation phenomena.

A different relationship was observed at the level of the pile base, where after achieving the maximum skin friction, the vertical stress in the soil still increased. This indicates that the increase was caused by the base of the pile, which was still far from a limit resistance.

The influence of the base resistance on the stress at the level of half pile depth is also shown in Figure 18c, where stress stabilization is observed. This phenomenon was not observed in Figure 18d because the stress in the soil and base resistance relationship is approximately linear.

**Figure 19:**
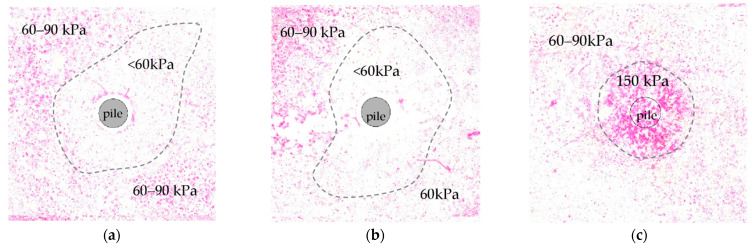
Pressure in the soil obtained from the test P6.R.CFS: (**a**) At half of the pile length level (20 cm below the ground). (**b**) At the pile base level (40 cm below the ground). (**c**) 10 cm below the pile base (50 cm below the ground).

Figure 19 shows the results from color pressure films placed horizontally in the soil at three different levels. The stresses presented in Figure 19a,b were too small to gain valuable outcomes because the tactile sensor’s stress measured in the previous investigation was less than 30 kPa. In contrast, the color pressure film 4 LW can measure the values in the range 50–200 kPa. Nevertheless, the result presented in Figure 19c is satisfactory because it shows the stresses distributed in circular shape with roughly a three times greater diameter than the pile one.

**Figure 20:**
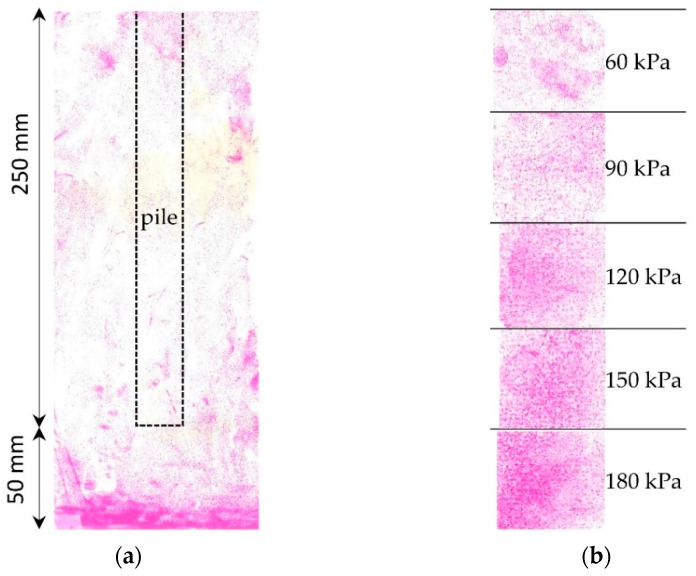
Pressure in the soil obtained from the test P5.R.CFS: (**a**) pressure map of horizontal stresses; (**b**) pressure scale.

In the test P5.R.CFS presented in Figure 20, the color film sensor 4 LW was placed vertically around the pile to measure horizontal stresses in soil. From this test, only the stresses below the pile base can be further analyzed because the horizontal stresses around the pile skin were out of the 4 LW color film range (50–200 kPa). Figure 20a presents the concentration of stresses 50 mm below the pile base. The value of the stresses are compared and presented in Figure 19c. The zone of high stresses below the pile base has a circular shape with a diameter of approximately 100 mm, so it is roughly three times the pile diameter. It confirms the soil behavior below the pile base investigated in the research of the soil displacements near the pile base [31].

**Figure 21:**
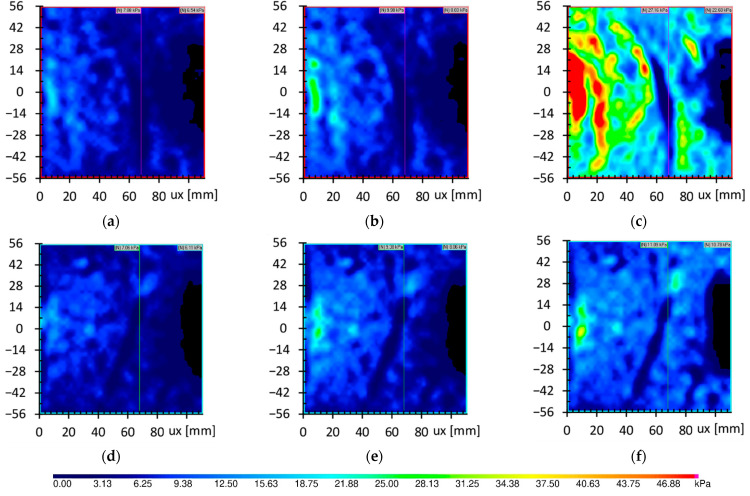
Pressure maps from test P1.R.0.5H.TS and P3.S.0.5H.TS in three different average stresses. Piles were placed in the middle of left maps edge, ux—distance from the pile skin. (**a**) P1.R.0.5H.TS-7.88 kPa; (**b**) P1.R.0.5H.TS–9.90 kPa; (**c**) P1.R.0.5H.TS–27.16 kPa; (**d**) P3.S.0.5H.TS-7.06 kPa; (**e**) P3.S.0.5H.TS–9.38 kPa; (**f**) P3.S.0.5H.TS–11.09 kPa.

**Figure 22:**
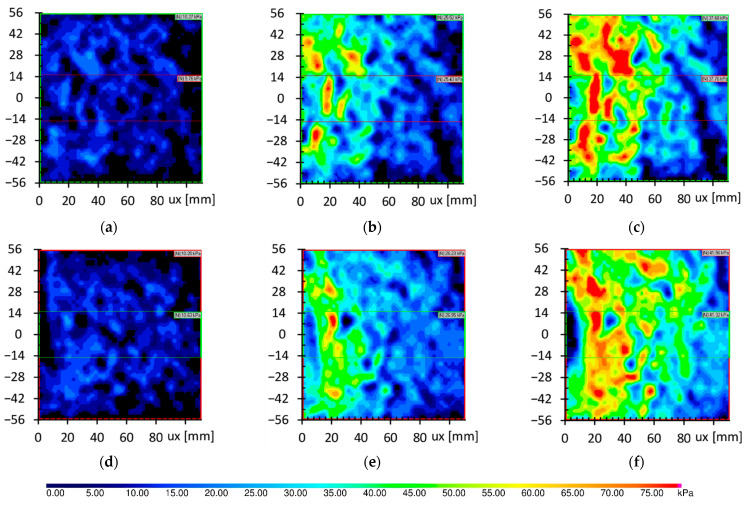
Pressure maps from test P2.R.H.TS and P4.S.H.TS with three different average stresses. The details of the piles are shown in Table 2. (**a**) P2.R.H.TS–10.27 kPa; (**b**) P2.R.H.TS–25.52 kPa; (**c**) P2.R.H.TS–37.68 kPa; (**d**) P4.S.H.TS–10.25 kPa; (**e**) P4.S.H.TS–26.23 kPa; (**f**) P4.S.H.TS–41.56 kPa.

Figure 21 and Figure 22 show the pressure maps placed at half of the pile depth and at the pile depth level, respectively. Piles touched the maps in the middle of the left edge in every map. Every map is presented for the chosen step of the pile load with the average pressure in the soil.

In the tests of P1.R.0.5H.TS and P3.S.0.5H.TS, where the tactile sensors were used for a rough and smooth pile, respectively, the maximum value of stress in soil occurred close to the pile skin and reduced due to the distance from pile skin. It is worth adding that the stress distribution for both rough and smooth piles presented in Figure 21a,b,d,e were initially comparable, as is presented widely in Figure 22. The sufficient friction can explain this in the interface between soil and pile skin in rough and smooth piles. Slipping the soil on the smooth pile in test P3.S.0.5H.TS caused the stress to reach a stable level finally. In contrast, the rough pile in test P1.R.0.5H.TS still had sufficient roughness to cause the further increase of stresses in the soil, as is presented in Figure 21c.

The pressure maps obtained in test P2.R.H.TS and P4.S.H.TS show that the stress concentration occurred not close to the pile, as in the case of test P1.R.0.5H.TS and P3.S.0.5H.TS, but in the space roughly equal 20 mm for pile base, as is presented in Figure 22. The results of both smooth and rough piles are very comparable to each other. The stresses at the base level reached greater values than the stresses at the level of half pile depth. Furthermore, the stresses near the pile base were reduced almost to zero when the applied load was close to the pile capacity, and a large pile settlement was observed. This reduction, presented in Figure 22f, might cause the degradation of friction conduction on pile skin close to the pile base. The stress reduction was more pronounced in the case of the smooth pile than in the rough pile. At high loads on the pile, the base of the pile still caused increasing vertical stresses, but only in the rough pile could it cause additional friction due to the increase in stresses.

**Figure 23:**
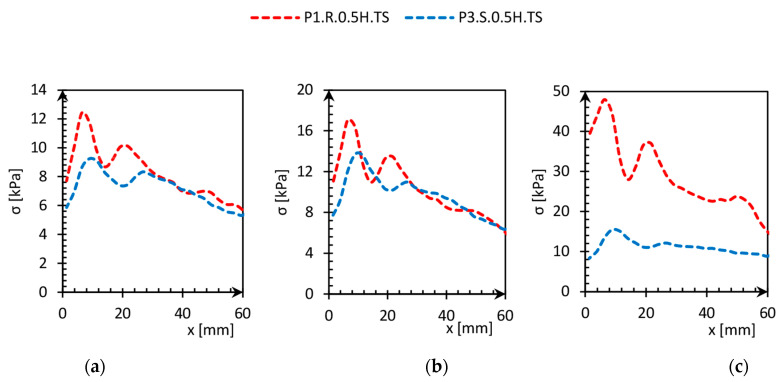
Stress redistribution versus distance from the pile skin (x) of P1.R.0.5H.TS and P3.S.0.5H.TS. (**a**) P1.R.0.5H.TS—7.88 kPa, P3.S.0.5H.TS—7.06 kPa; (**b**) P1.R.0.5H.TS—9.90 kPa, P3.S.0.5H.TS—9.38 kPa; (**c**) P1.R.0.5H.TS—27.16 kPa, P3.S.0.5H.TS—11.09 kPa.

**Figure 24:**
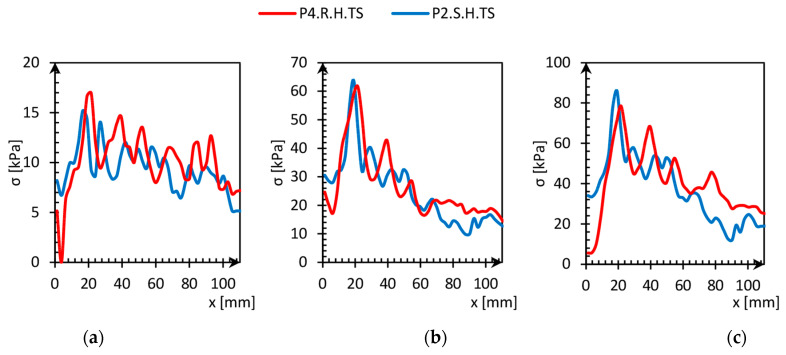
Stress redistribution versus distance from the pile skin (x) of P2.R.H.TS and P4.S.H.TS. (**a**) P2.R.H.TS—10.27 kPa, P4.S.H.TS—10.25 kPa; (**b**) P2.R.H.TS—25.52 kPa, P4.S.H.TS—26.23 kPa; (**c**) P2.R.H.TS—37.68 kPa, P4.S.H.TS—41.56 kPa.

Figure 23 and Figure 24 show the stress distribution in the surrounding soil due to the distance from the pile obtained based on the pressure maps. The stress in the soil surrounding the pile skin at the level of half pile depth reduces from a maximum value near the pile skin (3 mm) to geostatic stresses (6 kPa) in the distance equal to 60 mm, as presented in Figure 23. Stresses distribution of both smooth and rough piles in case of small loads were similar (Figure 23a,b), but the main difference can be noticed in Figure 23c, where only the rough pile still caused the stress to increase.

Stresses at a deeper level, presented in Figure 24, achieve greater values, and the stress distribution required more area to achieve geostatic pressure. It was out of the range of measurement during the test because of the dimensions of the tactile sensor (111 × 111 mm). Moreover, it can also be noticed that stress distribution at the pile depth level did not depend on the roughness of the pile skin.

## 6. Discussion

The results described in this paper indicate that stress mobilization in the soil at the pile depth level depends on the pile’s skin resistance in small loads when the slippage of soil on the pile skin had not yet occurred. After the maximum skin friction was achieved, only the stress at the level of pile depth increased. This increase might only be caused by the base of the pile that had not achieved the limit value. The stress increasing in the soil, which was caused by the pile skin resistance, could not only be caused by the dilatancy phenomena because stress increasing due to this process would be insufficient. It proves that the surrounding soil stresses that come from the pile skin and base resistance are interdependent, especially in the surrounding soil near the base of the pile. This phenomenon is usually neglected in pile load capacity analyzes because it is common to assume the skin and base resistance as independent components of pile load capacity. The pile base might increase the stress in the soil above the level of the pile base and cause additional skin resistance when the failure of skin friction does not occur. This positive relationship was observed in the rough pile. In the case of smooth pile skin, a large settlement of base caused the stress reduction in the soil close to the base of the pile, and thus, it reduced the skin friction.

In the present paper, two kinds of sensors were used, i.e., color film pressure and tactile sensors. Color film sensor 5 LW with a range of measuring of 6–41 kPa turned out to be too sensitive to measure the stress in soil, despite the laboratory conditions. A soft touch already caused a reading on the sensor, so it was hard to obtain valuable measurements. The stresses obtained in other sensors were promising and gained information about soil behavior during the pile load test, which is usually analyzed by the load-settlement curves [32,33]. This kind of sensor allows obtaining pressure maps that prevent skipping some areas in proper stress analysis and deeper investigation of pile–soil interaction.

The results presented in this paper proved that the tactile sensor used in this research could be successfully used to measure stress distribution in the soil in laboratory conditions or the case of small pile dimensions, for example, 3D printed concrete pile investigations [34].

## 7. Conclusions

This paper used flexible, thin sensors to measure the stresses in the soil surrounding the axially loaded smooth and rough model piles. Two sensors were used, i.e., color pressure film and tactile pressure sensor, with analog and digital measuring systems. Transferring stresses in soil caused by the piles still raises many doubts, and it is hard to measure in field and laboratory conditions. The novelty in this paper is tactile sensor applications that are not widely used in geotechnical research but may provide new insight into soil behavior and soil interaction with engineering structures. The results presented in this paper may be helpful in geotechnical sensor development.

The investigations carried out have led to the following conclusions:

- Tactile pressure sensors provide the maps of stress distribution in soil with high accuracy. The measuring is realized with frequency 1 Hz, which allows analyzing the stress in any stage of pile load. This sensor is also not affected by soil compaction.
- Color film sensors can be used as an additional measuring system to check the maximum pressure value in hard-to-reach places, because there is no equipment during the measuring.
- Calibration of the tactile sensor with soils with different particle sizes indicated that small grains caused a more uniform stress distribution than soil with coarse grains.
- Both skin and base resistance caused an increase in soil stress, and their influence was interdependent. Base resistance caused a stress increase not only below the base but also above the level of pile depth, simultaneously inducing additional stress, which improved the friction of the pile’s skin.
- The maximum stresses took place at 5 mm and 20 mm from the pile skin and the level of half pile depth and the level of pile depth, respectively.

## Figures and Tables

**Table 1:** Properties of piles.

Diameter, m	Length, m	Surface	Ra, µm
0.028	0.4	Rough	81.5
0.025	0.4	Rough	81.5
0.028	0.4	Smooth	5.08

**Table 2:** Type of sensors and localization used in pile load tests.

Pile	Type of Sensor	Location of Sensor
P1.R.0.5H.TS	Tactile sensor i-scan	Horizontally, depth equals half the height of the pile
P2.S.H.TS	Tactile sensor i-scan	Horizontally, depth equals the height of the pile
P3.S.0.5H.TS	Tactile sensor i-scan	Horizontally, depth equals half the height of the pile
P4.R.H.TS	Tactile sensor i-scan	Horizontally, depth equals the height of the pile
P5.R.CFS	Color film sensor	Horizontally, depth equals half the height of the pile, the height of pile, and 10 cm below the pile base ^1^
P6.R.CFS	Color film sensor	Vertically around the pile, 3.7 cm from the pile skin

^1^ Three sensors localizations.

## Data Availability

The data presented in this study are available within the article. They are also available on request from the corresponding author.
